# A meta-analysis of workplace exclusion on employee work behavior in the Chinese context

**DOI:** 10.3389/fpsyg.2025.1280074

**Published:** 2025-07-29

**Authors:** Zhengdong Li, Wenyu Li

**Affiliations:** ^1^School of Humanities, Shanghai Institute of Technology, Shanghai, China; ^2^Research Center for Organizational Behavior and Creative Management, Shanghai Institute of Technology, Shanghai, China; ^3^Antai College of Economics and Management, Shanghai Jiao Tong University, Shanghai, China

**Keywords:** workplace exclusion, positive work behavior, negative work behavior, Chinese context, meta-analysis

## Abstract

This study explores the impact of workplace exclusion on employee work behavior in the Chinese context. By employing the doctrine of the mean value orientation and self-consistency theory, the study aims to enhance the understanding of local workplace exclusion in China and its effects on employees’ work behavior. Furthermore, the study investigates the moderating role of employee types, data collection points, and data sources. Our meta-analysis included 24,662 participants from 72 independent samples. The findings indicate that workplace exclusion significantly influences employees’ positive work behavior negatively and their negative behavior positively. Moreover, the study identifies a moderating effect based on employee type (knowledge vs. non-knowledge type), revealing a stronger relationship between workplace exclusion and employee work behavior among knowledge employees. Examining the data collection point (cross-sectional vs. non-cross-sectional) as another moderating factor, the study demonstrates a stronger relationship between workplace exclusion and employee work behavior in the cross-sectional mode. Additionally, the data source (self-evaluation vs. superior evaluation plus self-evaluation) plays a moderating role, with employee self-evaluation strengthening the relationship between workplace exclusion and employee work behavior.

## 1 Introduction

Workplace exclusion poses a pervasive challenge in organizational settings. An illustrative incident that has gained recent attention involves employees facing ostracism simply for declining their leader’s toast. Workplace exclusion, encompassing various forms of mistreatment, such as physical, verbal, and psychological abuse, leads to a sense of exclusion and neglect among employees in the organization (Denise and Guy, 2020; Kandemir, 2025). This subjective experience is deeply felt by the victim (Fox and Stallworth, 2005; Ferris et al., 2008). Consequently, when individuals encounter exclusion in the workplace, their performance often declines, potentially jeopardizing the organization’s interests and hindering its overall development. As a response to this challenge, scholars have dedicated considerable attention to addressing workplace exclusion.

However, most current studies predominantly examine the impact of workplace exclusion on employee work behaviors within the Western cultural context (Hitlan and Noel, 2009; Khalid and Ahmed, 2016; Su et al., 2021). However, in different Chinese and Western cultural contexts, employees’ work behaviors and influence relationships are very different (Howard et al., 2020). Chinese organizational employees pay more attention to “social relations circle” and “face” (Du and Yao, 2015), and workplace exclusion extends to daily life, leading to even stronger negative perceptions. According to the logical framework of “event-cognition-behavior” (Lin et al., 2023), in the Chinese context, employees are more likely to suffer from workplace exclusion and generate more negative cognition, intensifying the damaging impact on their work behavior. Thus, workplace exclusion has its own unique performance in the Chinese context. Additionally, studies on the impact of workplace exclusion on employees’ work behaviors in the Chinese context have not reached a consistent conclusion (Su et al., 2021). Limited empirical methods in previous studies make it difficult to simultaneously measure and explore workplace exclusion on different employees’ work behaviors. Our research systematically clarifies the direction and intensity of the influence of workplace exclusion on employees’ work behavior, providing helpful guidance for managers to fulfill their organizational management role according to local conditions.

To address this gap, we aim to enhance our understanding of the influence of workplace exclusion on employee work behavior within the local Chinese context by introducing the doctrine of the mean, which is unique to China. The doctrine of the mean in China pertains to the shared cognition and values within a group influenced by collectivism (Du and Yao, 2015). In the Chinese cultural context, where collectivism is emphasized and there is a high perception of power distance (Hofstede, 1984), employees from higher organizational statuses may experience reduced self-esteem and value perception if excluded. Consequently, to avoid standing out, these employees may curtail their initiative. Conversely, within the Chinese cultural framework, personal relationships often intertwine with the workplace, forming various “relationship circles” or “factions” centered around common interests (Du and Yao, 2015). Workplace exclusion within these circles can lead to negative work behaviors, such as counter-productive behavior and deviant actions, especially when the exclusion is initiated by a close circle. The severity of harm suffered by excluded employees increases with the scale of such workplace exclusion. Furthermore, in the Chinese cultural context, where emotions are often concealed and effective communication is hindered by consideration of “face” and relationships, employees are more inclined to exhibit silent behavior (Halbesleben et al., 2014; Fitriastuti and Vanderstraeten, 2022). Against this backdrop of rapid changes in labor relations and continuous industrial structure upgrades in China, it is crucial to investigate the impact of workplace exclusion on employee work behavior.

To explore the factors influencing workplace exclusion and its impact on employee work behavior, we conducted a study examining the moderating effects of employee type, data collection point, and data source. Firstly, it was found that knowledge workers, heavily influenced by social norms and reliant on relationship networks for their work (Su et al., 2021), exhibit heightened sensitivity to workplace exclusion, likely experiencing a more significant impact on their work behavior. Secondly, we classified data collection time points into cross-sectional and non-cross-sectional designs. The former involves collecting data at a single time point, while the latter refers to longitudinal research that gathers data at two or more time points (Su et al., 2021). Employing various time points for data collection enables a more comprehensive understanding of employee cognition and behavior from a vertical perspective. Lastly, we categorized data sources into self-evaluation, superior evaluation plus self-evaluation (Wang et al., 2022). Studies indicated that individuals often perceive their own suffering more intensely(Gerber and Wheeler, 2009). Moreover, due to differing perspectives between superiors and employees, evaluations of workplace exclusion experienced by employees may diverge.

Our meta-analysis contributed significantly to the field in three key aspects. Firstly, while Su et al. (2021) utilized the meta-analysis method to identify the negative impact of workplace exclusion on employee attitudes and other aspects, they did not specifically analyze the effects of workplace exclusion on various work behaviors of excluded employees. In contrast, our study categorized different work behaviors and incorporated additional empirical literature, providing more robust evidence for the detrimental impact of workplace exclusion on employee work behaviors in the context of China. By synthesizing and revising the conclusions of each empirical study, we aim to minimize measurement errors in the process of variable measurement as much as possible (Wilson and Lipsey, 2000). Secondly, by differentiating the influence of employee types on workplace exclusion and employee work behavior, our analysis enhanced the understanding of distinct employee profiles and offered relevant practical management recommendations for each group. This differentiation allows for a more nuanced understanding of how workplace exclusion affects employees based on their individual characteristics. Lastly, we employed meta-analysis techniques to examine the moderating variables that might be impractical or challenging to measure in a single empirical study, such as the influence of workplace exclusion on employee working relationships. To achieve this, we carefully selected data collection time points and sources, aiming to overcome measurement limitations observed in existing studies. By integrating large-scale data, we derived more comprehensive and stable conclusions regarding the moderating variables and their impact on the relationship between workplace exclusion and employee work behavior.

## 2 Literature review and research hypothesis

### 2.1 The relationship between workplace exclusion and employees’ work behavior

According to Ferris et al. (2008), workplace exclusion takes various forms, categorized as either “hot” workplace violence (such as gossiping, verbal abuse, or physical attack) or “cold” workplace violence (such as indifference, avoiding eye contact, or social isolation). Regardless of its form, workplace exclusion directly affects employees’ fundamental needs: existence, belonging, self-esteem, and control (Williams, 2007). Consequently, this negative influence brings about changes in their work behaviors.

On one hand, the disregard and exclusion from the external environment leads to employees feeling marginalized by the organization (Robinson et al., 2013). In the Chinese context, being outside the social “circle” intensifies and diminishes employees’ sense of belonging, which in turn results in negative emotions like loneliness and anxiety. On the other hand, workplace rejection acts as a stressor for employees, impairing their cognitive processes (Dimotakis et al., 2011). Excluded employees not only hold negative evaluations toward other members of the organization, but also experience reduced self-perceptions, such as organizational self-esteem and the sense of work control (Cole et al., 2012). Consequently, we assert that in an organizational environment emphasizing harmony in Chinese culture, workplace exclusion directly undermines employees’ fundamental needs, leading to negative changes in their cognition and ultimately affecting their work behaviors.

Self-consistency theory can explain the relationship between workplace exclusion and employees’ work behavior. According to this theory, an individual’s external speech behavior is influenced by their motivation and cognitive consistency (Korman, 1970). When an individual’s cognition changes regarding something, they tend to take actions that align with their own interests (Mulder and Aquino, 2013). Employee work behavior includes a variety of actions undertaken by individuals in an organization to ensure its normal operation and effectiveness (Peng et al., 2016). Consequently, workplace exclusion can result in cognitive impairment, which in turn diminishes employees’ motivation to contribute to the organization’s smooth functioning. As a result, their work effort and positive work behaviors are reduced, reflecting their dissatisfaction. Moreover, workplace exclusion in the Chinese context extends beyond the workplace, exacerbating employees’ negative cognition and disrupting their everyday living space. This, in turn, fosters negative work behaviors that impede the organization’s proper functioning.

Workplace exclusion can hinder positive work behaviors, including organizational citizenship behavior, initiative behavior, and voice behavior. Some scholars categorize employees’ work behaviors into different domains: attraction to the organization, active engagement with the organization, completion of assigned tasks, and undertaking out-of-role behaviors (Katz and Kahn, 2015). Extra-role behavior encompasses spontaneous actions that extend beyond the formal reward system but benefit the organization (Organ, 1988). Organizational citizenship behavior, initiative behavior, and voice behavior, all falling under the umbrella of extra-role behavior, are considered as positive work behaviors. Being dependent on employees’ internal driving forces, extra-role behavior is susceptible to external interference. Workplace exclusion, considered a form of workplace violence, has a higher likelihood of reducing employees’ work motivation and evoking negative emotions, which consequently hampers positive work behaviors.

Similarly, workplace exclusion can elicit negative behaviors, including counterproductive behavior, silent behavior, deviant behavior, and pro-organizational unethical behavior. The self-consistency theory emphasizes the alignment between internal motivation and external behavior. When employees experience exclusion and rejection from their colleagues, negative self-perceptions emerge (Gerber and Wheeler, 2009). Consequently, these employees may feel inferior and marginalized. To demonstrate their loyalty, they may even engage in pro-organizational and non-ethical behavior. Furthermore, this negative self-perception can hinder employees’ performance in both their work and social lives. Driven by a desire for revenge, employees may intensify negative work behaviors, such as counterproductive and deviant actions, to express their discontent. Moreover, adhering to the mean value orientation of collectivism, excluded employees often choose to display outward friendliness instead of directly communicating with others due to considerations of “face” (Du and Yao, 2015). Unfortunately, this further exacerbates the alienation among employees and consequently increases silent behavior. Based on these observations, we propose the following hypothesis:

*Hypothesis* 1: Workplace exclusion inhibits employees’ positive work behaviors such as organizational citizenship behavior, initiative behavior, and voice behavior.

*Hypothesis* 2: Workplace exclusion reinforces employees’ negative work behaviors including counter-productive behaviors, silent behaviors, deviant behaviors, and pro-organizational unethical behaviors.

### 2.2 The relationship between workplace exclusion and employees’ work behavior: moderating effect

Differences in employee types can be categorized into knowledge workers and non-knowledge workers. Knowledge workers, who typically hold higher educational qualifications, utilize knowledge and information in their work, such as information research and development, internet operations, financial securities, and government agencies (Drucker, 1979). On the other hand, non-knowledge workers have lower educational backgrounds and are involved in manual labor, including migrant workers, frontline employees in the service industry (e.g., hotels), and frontline workers in manufacturing industries (Su et al., 2021).

There are variances between knowledge workers and non-knowledge workers in terms of values and skills required for their work (Marrone and Carson, 2007). Firstly, knowledge workers possess extensive knowledge and skills due to their higher education levels. They collaborate closely with other members within the organization to achieve common goals (Zhan et al., 2013). Secondly, knowledge workers prioritize spiritual growth and self-fulfillment, while non-knowledge workers place more emphasis on material income and fulfilling basic needs (Crawford and Lepine, 2013). Thus, it can be inferred that individuals with higher levels of education tend to perceive their organizational status as more important. In the Chinese context, it is crucial for employees to strive for a balanced and harmonious relationship within the organization. Consequently, knowledge workers, compared to non-knowledge workers, demonstrate a heightened consciousness toward workplace exclusion and its adverse consequences. Subsequently, when confronted with workplace exclusion, knowledge workers are more likely to display a decline in positive work behaviors while exhibiting an increase in negative work behaviors. Hence, based on these observations, we put forth the following hypothesis:

*Hypothesis* 3: Employee type (knowledge vs. non-knowledge type) can moderate the relationship between workplace exclusion and employee work behavior. Specifically, the relationship between workplace exclusion and employee work behavior is stronger among knowledge workers compared to non-knowledge workers.

Data collection point difference: According to the time point of data collection, the cross-sectional design can be divided into two categories: cross-sectional design and non-cross-sectional design. Cross-sectional design refers to data collection at the same time point, while non-cross-sectional design refers to longitudinal research that collects data at two or more time points (Chen et al., 2016). In the context of employee data collection, a non-cross-sectional design allows for a vertical understanding of employees’ cognition and behavior (Du and Yao, 2015). The harm caused by workplace exclusion to employees is a continuous process. Due to the unique Chinese value orientation of collectivism, employees are influenced to maintain a friendly and harmonious relationship with other members of the organization (Du and Yao, 2015). As a result, employees may perceive marginalization more intensely in the short term, but its negative impact diminishes over the long term. Based on this, the study concludes that the relationship between workplace exclusion and employees’ work behavior is stronger in a cross-sectional design study. Consequently, we propose the following hypothesis:

*Hypothesis* 4: The time point of data collection (cross-sectional vs. non-cross-sectional) can regulate the relationship between workplace exclusion and employee work behavior, with the relationship being stronger in cross-sectional data than in non-cross-sectional design.

The variation in data sources plays a crucial role in the empirical scale, which can be categorized into self-evaluation and other evaluation. Self-evaluation refers to the method where employees independently judge and provide free answers to the scale’s content, while other-evaluation involves organization members honestly judging and answering based on employees’ working conditions (Wang et al., 2022; Paulhus and Reynolds, 1995). The evaluation results of workplace exclusion are expected to vary due to the differing perceptions between superiors and employees. Even if the research designs and conditions remain constant, different evaluation methods yield distinct outcomes (Carpenter et al., 2014). Generally, individuals have a better understanding of their own experiences. Furthermore, influenced by China’s collectivist value orientation, employees tend to develop superficial friendships with their colleagues within the organization. As a result, leaders perceive a lower level of workplace rejection among subordinates. This in turn reduces the impact of perceived workplace rejection on employee work behavior. It follows that when relying on self-rated data sources, the relationship between workplace exclusion and employee work behavior becomes more pronounced. Consequently, we propose the following hypothesis:

*Hypothesis* 5: The data source (self-evaluation vs. superior evaluation plus self-evaluation) has the ability to regulate the relationship between workplace exclusion and employee work behavior. Specifically, the relationship between workplace exclusion and employee work behavior is stronger when self-appraisal data sources are employed compared to superior appraisal and self-appraisal matching.

**Figure 1:**
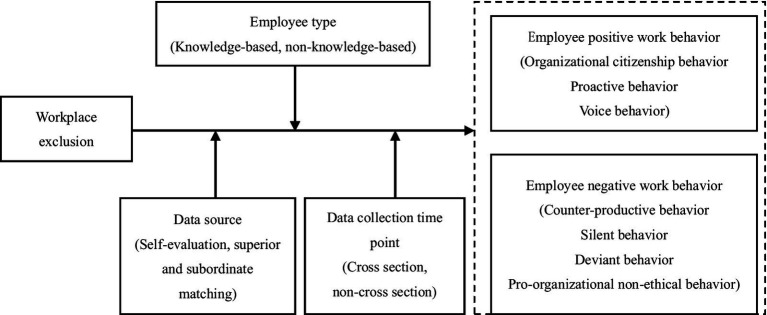
Hypothetical model.

Based on the above, the model built in this study is shown in Figure 1.

## 3 Research methods

### 3.1 Literature retrieval, screening and coding

#### 3.1.1 Literature search

In order to investigate the correlation between workplace exclusion and employee work behavior, we conducted a meta-analysis. We utilized the keywords “workplace exclusion,” “exclusion behavior,” “marginalization,” and “exclusion” to search for relevant Chinese literature in databases such as the China Excellent Master’s Dissertation Database, Doctoral Dissertation Database, CNKI, Wanfang Data, VIP, among others. Additionally, we employed the keywords “Workplace Ostracism,” “Exclusion Behavior,” and “Job Exclusion” to retrieve English literature from reputable databases including Web of Science, Sage, and Wiley. After conducting a thorough search, it was identified that empirical research on the correlation between workplace exclusion and employees’ work behavior in the Chinese context first emerged in 2010. Therefore, for our study, the literature search period extends from 2010 to December 31, 2022.

#### 3.1.2 Inclusion criteria

**Figure 2:**
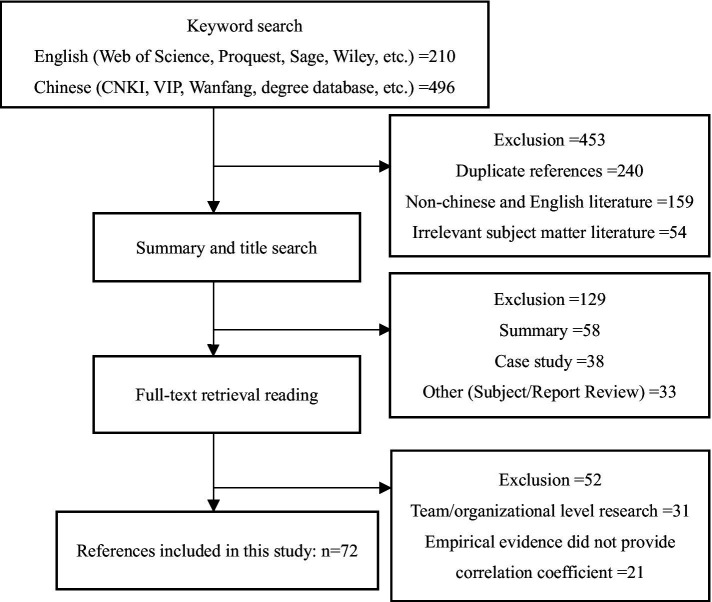
Screening process.

As depicted in Figure 2, we selected academic dissertations and journal articles based on the following inclusion criteria: (1) Empirical studies focusing on the relationship between workplace exclusion and individual work behaviors of employees, excluding non-empirical studies such as cases. (2) Studies explicitly reporting the correlation coefficient and sample effect value of workplace exclusion and work behavior, excluding those utilizing structural equation models and other statistical methods. (3) Survey data entered in this study will not be reused. If the same sample is published in multiple literatures, it will be considered as one study (Byron et al., 2010). (4) The sample consisted of employees in Chinese organizations, within the context of the workplace. (5) The focus of the study is on the phenomenon of workplace exclusion at the individual level, excluding literature exploring team and organizational levels. (6) The coding content includes the title of the literature, journal, year, author, type of employee’s work behavior, and the correlation between variables.

Two coders coded the included articles and exchanged the results for review, with a high consistency rate of 98.6% for the encoding process. The main outcome variables were categorized as employee positive work behavior and employee negative work behavior. Ultimately, a total of 72 empirical studies were included, consisting of 62 Chinese literature and 10 English literature sources, which collectively involved 24,662 participants. The detailed final selection process can be observed in Figure 2.

### 3.2 Meta-analysis program

We used the Metafor package in R4.2.0 to calculate the results, employing the Pearson correlation coefficient (r) as the effect size. We applied the Hunter-Schmidt meta-analysis method, which is commonly used in management research (Hunter and Schmidt, 2004). We conducted tests for publication bias, heterogeneity, main effects, and moderating effects.

Publication bias testing is conducted to assess the accuracy of the included meta-analysis literature. We utilized Egger’s regression coefficient (Egger and Smith, 1997), Begg rank correlation test (Begg and Berlin, 1988), and the loss of safety coefficient to evaluate publication bias effects among variables. If the *p*-value of Egger’s regression coefficient and the p-value of the Begg rank correlation test are not statistically significant (*p* > 0.05), it indicates the absence of publication bias. Additionally, the loss of safety factor focuses on determining the number of unpublished studies needed to rule out publication bias. If the loss of safety factor does not exceed 5 K + 10, the research may be considered free from publication bias.

Next, we explored the heterogeneity test of meta-analysis. The included studies in the meta-analysis differ in empirical design, survey objects, sample size, results, and other factors, leading to heterogeneity. Heterogeneity among different effect sizes is also present. The test results of the heterogeneity of effect size determine whether a fixed effect model or random effect model is suitable for further research. In this study, we evaluated the heterogeneity of the sample through the test results of the Q statistic and I^2^ value. The Q test reflected the statistical significance (*p* < 0.001) of workplace exclusion of employees and outcome variables. The critical points of 25, 50, and 75% for the I2 value represented low, medium, and high degrees of heterogeneity, respectively (Higgins et al., 2002). Lastly, we reported Q, I^2^, Tau, Tau2, and the corresponding *p*-values for heterogeneity.

Finally, we reported the analysis results of the main effect and the moderating effect. The main effect test considered variables such as effect size K, sample size N, weighted average correlation coefficient r, and a 95% CI confidence interval. A 95% confidence interval that excludes zero indicates that the corrected correlation is statistically significant (Higgins et al., 2002). For the moderating effect, we examined the strength of the influence of each moderating variable on employee work behavior. We began by coding employee types, cultural backgrounds, and variable measures. Knowledge workers are predominantly employed in fields such as information research and development, internet, finance and securities, and government organizations, while non-knowledge workers are mainly employed in manual labor, service industries, and manufacturing industries (Su et al., 2021). To ensure research rigor, we only accepted studies with explicit industry sources. The time point of the data used in the paper was also taken into account.

## 4 Results

### 4.1 Publication bias test

**Figure 3:**
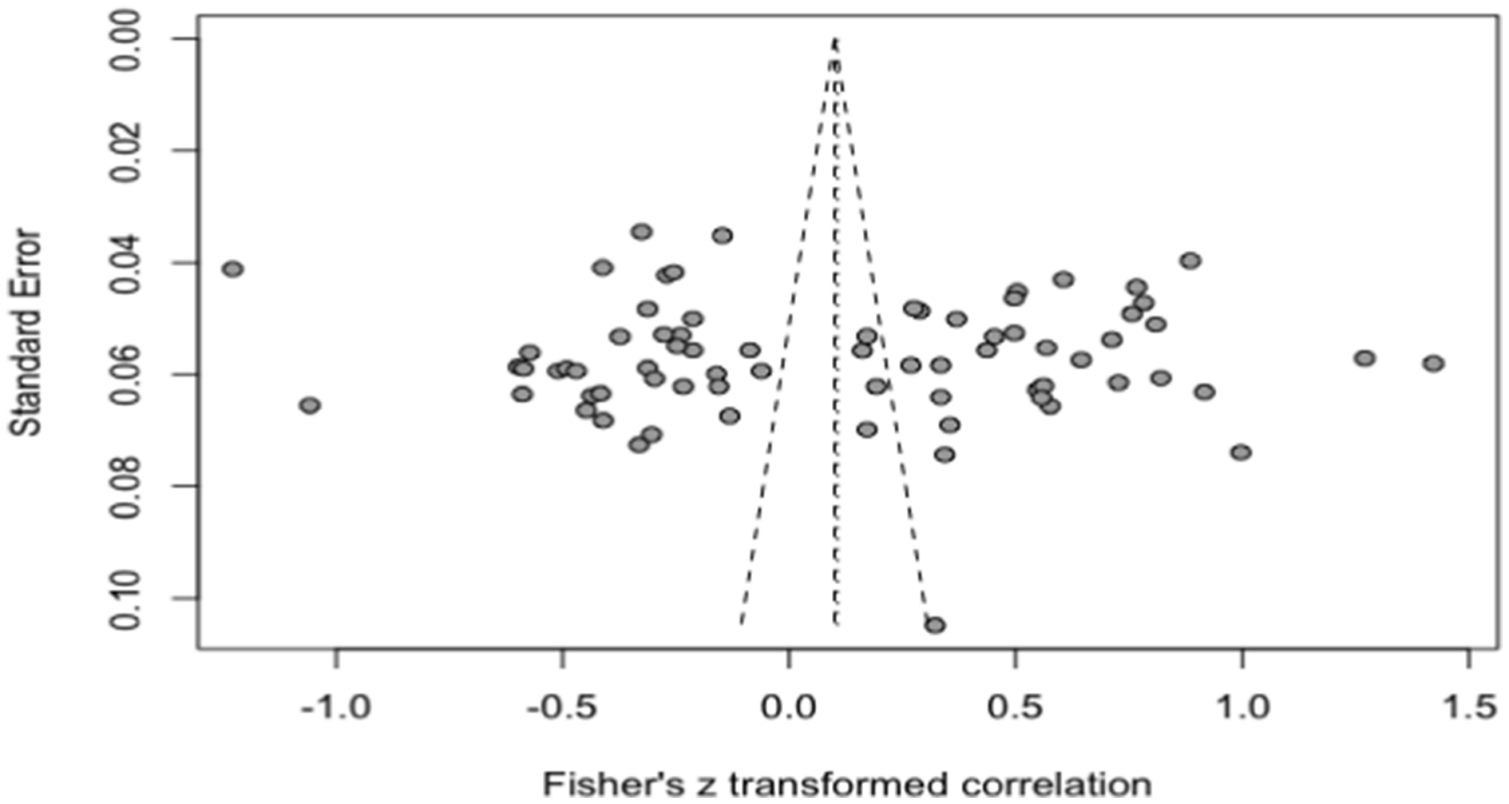
Funnel diagram of effect value distribution of the overall relationship between workplace exclusion and work behavior.

Publication bias refers to the phenomenon wherein studies with statistically significant results are more likely to be acknowledged and published within the academic community compared to studies with non-statistically significant results (Begg and Berlin, 1988). Consequently, the published literature is unable to encompass the entirety of completed research. To address this issue, our paper employs a combination of qualitative and quantitative methods. Initially, a funnel plot is utilized to visually assess the publication bias with regards to workplace exclusion and its impact on employees’ overall work behavior. Additionally, we employ Egger’s regression coefficient, Begg rank correlation test, and the insecurity coefficient as indicators for further analysis. Figure 3 demonstrates that the effect size of the relationship between job field exclusion and employee work behavior in the included studies exhibits a symmetrical distribution around the average effect size. This indicates a lack of substantial publication bias within the included studies.

As shown in Table 1, the results indicate that there is no significant relationship between workplace exclusion and positive work behavior (*p* = 0.936), organizational citizenship behavior (*p* = 0.577), proactive behavior (*p* = 0.230), voice behavior (*p* = 0.519), and negative work behavior (*p* = 0.519). Similarly, there is no significant relationship between workplace exclusion and Egger’s (0.409), counter-productive behavior (*p* = 0.687), silent behavior (*p* = 454), deviant behavior (*p* = 0.435), and pro-organizational non-ethical behavior (*p* = 0.428), suggesting the absence of publication bias. The Begg rank correlation tests also reveal no significant relationship between workplace exclusion and positive work behavior (*p* = 0.287), organizational citizenship behavior (*p* = 0.626), proactive behavior (*p* = 0.348), voice behavior (*p* = 0.221), negative work behavior (*p* = 0.505), and counter-productive behavior (*p* = 0.221). Likewise, the Begg rank correlation tests show no significant relationship between workplace exclusion and silent behavior (*p* = 453), deviant behavior (*p* = 0.327), and pro-organizational unethical behavior (*p* = 0.602), further supporting the absence of publication bias. Lastly, the coefficient of loss of safety, which exceeds 5 K + 10 for all workplace exclusion and outcome variables, confirms the stability of the study’s conclusion and the absence of significant publication bias.

**Table 1:** Publication bias test for workplace exclusion.

Result variable	Egger’s regression coefficient test		Begg rank correlation test		Fail-safe factor	
	*Intercept*	*p*	*z*	*p*	*Nfs*−0.05	5 *K* + 10
Job positive behavior	−0.358	0.936	−1.070	0.287	38,057	185
Organizational citizenship behavior	−0.625	0.577	−0.490	0.626	18,484	110
Proactive behavior	0.048	0.230	−0.940	0.348	447	40
Voice behavior	−0.085	0.519	−1.220	0.221	40	25
Negative work behavior	0. 808	0.409	−0.670	0.505	120,658	195
Counter-productive behavior	0.075	0.687	0.960	0.337	16,862	70
Silent behavior	1.185	0.454	−0.750	0.453	3,030	45
Deviant behavior	2.072	0.435	−0.980	0.327	1,181	35
Pro-organizational non-ethical behavior	1.463	0.428	−0.520	0.602	301	25

*p* is the regression coefficient; *z* is two-tailed test; Nfs−0.05 indicates the failure factor.

### 4.2 Main effect and heterogeneity test

In the relationship between workplace exclusion and employee work behavior, only the effect sizes of voice behavior were found to be non-significant (Q = 1.520, *p* > 0.005, I^2^ = 0.000), indicating a lack of heterogeneity and therefore the adoption of the fixed-effect model. Additionally, both the Q and I^2^ values for the relationship between workplace exclusion and its outcome variables satisfied the conditions for heterogeneity, necessitating the use of the random effects model. For instance, the effect values of workplace exclusion and employee positive behavior displayed an I^2^ value of 95.800, suggesting that 95.800% of the observed difference can be attributed to differences in effect values, while random error accounts for 4.2000% of the observed difference. Tau2, which represents the ratio of inter-study variation used for weight calculation (Carpenter et al., 2014), is equal to 0.056, indicating that 5.6% of the inter-study variation can be used in weight calculation.

The effect values of the relationship between workplace exclusion and employees’ positive work behavior and negative work behavior are displayed in Table 2. Notably, workplace exclusion demonstrates significant effect sizes when correlated with employee active work behavior (*r* = −0.376), organizational citizenship behavior (*r* = −0.409), initiative behavior (*r* = −0.313), and voice behavior (*r* = 0.166), with 95% confidence intervals that do not encompass zero. Likewise, workplace exclusion also exhibits significant effect sizes when linked to negative work behavior (*r* = 0.566), counterproductive behavior (*r* = 0.545), silent behavior (*r* = 0.573), deviant behavior (*r* = 0.542), and pro-organizational unethical behavior (*r* = 0.361), with 95% confidence intervals that do not encompass zero. The research findings indicate that, apart from the moderately negative correlation between workplace exclusion and voice behavior, all other correlation coefficients surpass 0.3, indicating a significantly strong correlation between them (Gignac and Szodorai, 2016). Consequently, it can be concluded that workplace exclusion exerts a significant impact on both positive and negative work behaviors of employees. Hence, both hypotheses 1 and 2 are supported.

**Table 2:** Main effect test results and heterogeneity test results of the relationship between workplace exclusion and employee work behavior.

Result variable	*K*	*N*	Model	Homogeneity test						Main effect analysis				
				*Q*	*df(Q)*	*p*	*I^2^*	*Tau*	*Tau^2^*	Point estimates and 95%CI			Two-tailed test	
										*r*	Floor	Upper limit	*z*	*p*
Employee positive behavior	35	12,376	R	801.92	34	0.000	95.800	0.236	0.056	−0.376	−0.457	−0.296	−9.17	0.000
Organizational citizenship behavior	20	7,214	R	673.58	19	0.000	97.200	0.293	0.086	−0.409	−0.540	−0.278	−6.140	0.000
Proactive behavior	6	1846	R	13.840	5	0.017	63.900	0.078	0.006	−0.313	−0.391	−0.234	−7.830	0.000
Voice behavior	3	1,336	F	1.520	2	0.467	0.000	0.000	0.000	−0.166	−0.220	−0.112	−6.030	0.000
Employee negative behavior	37	12,286	R	966.630	36	0.000	96.300	0.290	0.084	0.566	0.470	0.660	11.640	0.000
Counter-productive behavior	12	4,092	R	548.470	11	0.000	98.000	0.402	0.162	0.545	0.316	0.775	4.650	0.000
Silent behavior	7	2031	R	69.460	6	0.000	91.400	0.192	0.037	0.573	0.424	0.722	7.540	0.000
Deviant behavior	5	1,205	R	52.810	4	0.000	92.400	0.227	0.052	0.542	0.335	0.749	5.130	0.000
Pro-organizational non-ethical behavior	3	1,275	R	36.021	2	0.000	93.230	0.156	0.024	0.361	0.177	0.544	3.847	0.000

k is the number of effect values; N is the number of independent samples; R is the random effects model; F is the fixed-effect model; Q is the homogeneity test; df(Q) is the degree of freedom; *p* is the significance level; I2 is the proportion of the true difference in effect size to the observed variation; r is the meta-analysis estimate; z is the statistic of the two-tail test.

### 4.3 Adjustment effect test

As demonstrated earlier, there is heterogeneity in the effect size of the relationship between workplace exclusion and employees’ positive and negative work behavior. Hence, the random effects model is applied to examine the moderating effects of employee type, data collection time point, and data source. Since there is limited literature available on each dimension of employee work behavior, this study focuses on testing the overall moderating effect of workplace exclusion on employee positive behavior, employee negative behavior, and employee type. Additionally, it explores potential differences in the impact of workplace exclusion on employees’ work behavior based on categories such as knowledge workers vs. non-knowledge workers, cross-sectional design vs. non-cross-sectional design, and employee self-evaluation vs. evaluation by superiors and subordinates.

Table 3 presents the results. Based on the moderating effect of employee type, the relationship between workplace exclusion and employee work behavior is found to be significantly influenced by employee type. Specifically, employee type significantly affected the relationship between workplace exclusion and employee positive behavior (Qb = 14.16, *p* < 0.001). It was observed that the relationship between employee exclusion and positive behavior was significantly stronger among knowledge workers (*r* = −0.577) compared to non-knowledge workers (*r* = −0.166). Employee type also significantly affected the relationship between workplace exclusion and employee negative behavior (Qb = 8.56, *p* = 0.003). The relationship between workplace exclusion and negative behavior among knowledge employees (*r* = 0.740) was significantly higher than for non-knowledge employees (*r* = 0.367). Therefore, hypothesis 3 is supported.

**Table 3:** Test results of adjustment effect.

Regulating variable	Work behavior	*Qb*	*df*	*p*	Variable class	*k*	*N*	Point estimates and 95% CI		
								*r*	Floor	Upper limit
Employee type	positive	14.16	1	0.000	knowledge-based	5	2,962	−0.459	−0.577	−0.340
					nonknowledge-based	3	1,016	−0.166	−0.262	−0.070
	passive	8.56	1	0.003	knowledge-based	3	1,111	0.740	0.649	0.831
					nonknowledge-based	3	962	0.367	0.134	0.600
Data collection time point	positive	19.75	1	0.000	Cross section	12	5,322	−0.615	−0.763	−0.467
					Non-cross section	7	5,559	−0.242	−0.314	−0.170
	passive	65.56	1	0.000	Cross section	19	8,157	0.760	0.652	0.868
					Non-cross section	7	8,139	0.251	0.192	0.310
Data source	positive	14.94	1	0.000	self-assessment	15	8,784	−0.545	−0.680	−0.411
					Superior and subordinate matching	14	10,081	−0.247	−0.316	−0.178
	passive	29.97	1	0.000	self-assessment	20	7,998	0.732	0.624	0.841
					Superior and subordinate matching	4	1968	0.352	0.270	0.434

Qb is homogeneity test; See Table 2 for others.

Regarding the moderating effect of data collection time point, the results indicate that the time point of data collection significantly influences the relationship between workplace exclusion and employees’ work behavior. Specifically, the data collection time point significantly affected the relationship between workplace exclusion and employee positive behavior (Qb = 19.75, *p* < 0.001). The relationship between workplace exclusion and positive behavior was found to be significantly higher under the cross-sectional design (*r* = −0.615) compared to the non-cross-sectional design (*r* = −0.242). Furthermore, the data collection time point significantly affected the relationship between workplace exclusion and employee negative behavior (Qb = 65.56, *p* < 0.001). The relationship between workplace exclusion and negative behavior was considerably higher in the non-cross-sectional design (*r* = 0.760) compared to the cross-sectional design (*r* = 0.251). Hence, hypothesis 4 is supported.

The relationship between workplace exclusion and employees’ work behavior is significantly influenced by the moderating effect of data sources such as self-rating and superior rating, as well as self-rating matching. Specifically, the method of data collection from different sources has a significant impact on the link between workplace exclusion and employee positive behavior (Qb = 14.94, *p* < 0.001). The strength of the relationship between workplace exclusion and employee positive behavior (*r* = −0.545) was found to be significantly stronger than the relationship observed when using the design involving superior evaluation and self-evaluation (*r* = −0.247). Similarly, different data sources significantly influenced the relationship between workplace exclusion and employees’ negative behaviors (Qb = 29.97, *p* < 0.001). Under the specific condition of using self-assessment as the data collection method, the relationship between workplace exclusion and employees’ negative behaviors (*r* = −0.732) was found to be significantly stronger than the relationship between workplace exclusion and employees’ positive behaviors when utilizing the matching design of superior evaluation and self-evaluation (*r* = 0.352). Therefore, hypothesis 5 has been confirmed.

## 5 Discussion

Applying the doctrine of the mean value orientation and utilizing self-determinism theory, our meta-analytical review examined various relationships between workplace exclusion and distinct work behaviors exhibited by employees. Our analysis, based on a comprehensive review of empirical studies, largely aligned with the theoretical model depicted in Figure 1. Specifically, we identified nine pairs of significant bivariate relationships, indicating that workplace exclusion significantly influences diverse work behaviors.

Furthermore, our study delved into the moderating effects of employee type, data collection time point, and data source. Our findings affirmed the original theoretical hypothesis, suggesting that workplace exclusion exerted a more pronounced impact on employee work behavior under specific conditions. Notably, the boundary conditions that amplified this impact included knowledge workers, a cross-sectional design, and a self-evaluation method.

### 5.1 Theoretical significance

Our meta-analysis, encompassing 24,662 participants across 72 samples, yielded three primary theoretical implications. Firstly, examining the behavior pattern of the excluded, we systematically elucidated the relationship between workplace exclusion and both positive and negative work behaviors among employees in the Chinese context. In essence, workplace exclusion exerted varying degrees of damage to a spectrum of work behaviors. Our study, more extensive than those of Howard et al. (2020) (K = 58) and Li et al. (2021) (K = 35), established a more reliable and stable foundation for subsequent research.

Secondly, In the study of the impact of workplace exclusion on employees, we focus on the employee characteristics in terms of mean value orientation, expanding on previous studies that primarily focused on the explanatory mechanism of social identity (Halbesleben et al., 2014; Kandemir, 2024). The research conclusion indicates that workplace exclusion has varying degrees of destructive effects on different types of employees. Among them, knowledge employees are more concerned about organizational harmony and thus are more sensitive to the perception of workplace exclusion. This highlights the importance of distinguishing employee types under the influence of the mean idea in this study.

Thirdly, our results clarify the moderating effect of two measurement factors: the time point of data collection and data source. This helps address the measurement limitations that cannot be resolved in a single empirical study. Our findings demonstrate that workplace exclusion has a greater negative impact on employees when the measures used in empirical studies come from cross-sectional designs or self-rated data. In other words, studies that fail to account for common method bias show stronger correlations, underscoring the importance of addressing common method bias in empirical research. Through the above analysis, this study thoroughly elucidated the internal mechanisms underpinning the influence of workplace exclusion on employee work behavior in the Chinese context.

### 5.2 Practical significance

Firstly,our research has identified the negative impact of workplace exclusion on employee behavior. To address this issue, managers should consider reevaluating corporate values and organizational culture. It is important for organizations to foster a goal-oriented and friendly culture, which promotes cohesion and provides social and emotional support to enhance employee identification. In the Chinese context, it is essential for managers to monitor informal organizational values, such as the “small circle” gang culture, as a means to cultivate fair competition.

Secondly, the results of our study indicate that excluded employees have immediate negative cognitive responses, and cognition serves as a key prerequisite for behavior change (Jahanzeb and Fatima, 2018). Therefore, it is crucial for organizational managers to intervene promptly when employees hold negative perceptions. Managers should prioritize monitoring emotional changes in employees and offer timely assistance to those who are excluded. Excluded employees should also take the initiative to seek help and support from the appropriate individuals.

Thirdly, our findings demonstrate that knowledge workers are more susceptible to perceiving workplace rejection due to their higher expectations of self-actualization and organizational identity (Crawford and Lepine, 2013; Stamper et al., 2025). When designing the compensation mechanism, organizations should adopt a dynamic approach, providing different material incentives and emotional support based on individual employees. This ensures that the compensation mechanism maximizes its effectiveness.

### 5.3 Future research direction

This study conducted a meta-analysis based on the perspective of excluded employees, concentrating solely on the adverse effects of workplace exclusion on them. However, exploring the motivations behind exclusionists’ desires to exclude others is essential for mitigating workplace exclusion at its source. Therefore, a shift in perspective is necessary to delve into the mechanism of workplace exclusion.

Secondly, this paper exclusively focuses on the impact of workplace exclusion on work behavior in the Chinese context, without a separate meta-analysis in the Western context. Consequently, it fails to comprehensively and systematically differentiate employees’ subsequent behavioral decisions under cultural differences. This aspect could be explored further in future research.

In addition, our study investigates the moderating effect of measurement factors. Our findings demonstrate that the presence of common method bias can introduce bias into research outcomes. Therefore, it is crucial for future scholars to give due consideration to sample sources while designing experiments and selecting variables.

Furthermore, there is a significant relationship between employees’ different work behaviors and their intrinsic motivation (Korman, 1970). However, due to a limited number of empirical studies, an in-depth investigation could not be carried out. As future studies mature, psychological variables such as cognition and motivation can be considered to further explore the mechanism of workplace exclusion on employee work behavior.

### 5.4 Limitations

Regarding the study’s shortcomings, firstly, from a structural perspective, workplace exclusion can be categorized into superiors’ exclusion, colleagues’ exclusion, and language exclusion (Ferris et al., 2008). Future studies could delve into these subcategories to explore the varying relationship strengths between workplace exclusion and employee behavior in different dimensions.

Secondly, due to the focus on the Chinese context, the limited number of effect values included in the meta-analysis raises concerns about the accuracy of the research. The study’s validity can be further tested and analyzed once future academic research provides more data.

Thirdly, the current research mainly focuses on employee type and empirical research design as moderating variables. However, social approval bias is also a crucial factor influencing evaluation results and should be considered in future investigations (Crowne and Marlowe, 1960).

## 6 Conclusion

In this study, we developed a meta-analysis model to investigate the impact of workplace exclusion on both positive and negative work behaviors among employees in the Chinese context. Furthermore, we explored the moderating effect of employee type (knowledge vs. non-knowledge type) on the relationship between workplace exclusion and employee work behavior. Additionally, we scrutinized the moderating effects of measurement methods, specifically examining the data collection point (cross-sectional vs. non-cross-sectional) and data source (self-evaluation vs. superior-evaluation plus self-evaluation) in relation to workplace exclusion and employee work behavior. The objective of employing meta-analysis, as a comprehensive and systematic analysis method, is to offer a stable, reliable, and clear direction for future research. We aspire that future researchers will continue to delve into the mechanism through which workplace exclusion influences employees’ work outcomes in the Chinese context.

## Data Availability

The original contributions presented in the study are included in the article/supplementary material, further inquiries can be directed to the corresponding author.
